# Cost-effectiveness of the CV-polypill strategy versus standard care for secondary cardiovascular prevention in Spain: an analysis based on the SECURE trial

**DOI:** 10.1016/j.lanepe.2025.101348

**Published:** 2025-06-27

**Authors:** Thomas Gaziano, José María Castellano, Amy Dymond, Alissa Looby, Stuart Mealing, Ruth Owen, Stuart Pocock, José Ramón González-Juanatey, Alberto Cordero, Antonio Fernández-Ortiz, Aleš Linhart, François Schiele, Wolfram Doehner, Luisa Ojeda Fernández, Valentín Fuster

**Affiliations:** a Brigham and Women's Hospital, Harvard Medical School, Boston, MA, USA; b Centro Nacional de Investigaciones Cardiovasculares (CNIC), Madrid, Spain; c York Health Economics Consortium (YHEC), York, United Kingdom; d London School of Hygiene and Tropical Medicine, London, United Kingdom; e Hospital Clínico Universitario de Santiago de Compostela, IDIS, CIBERCV, Santiago de Compostela, Spain; f Cardiology Department, Hospital Universitario de San Juan, Alicante, Spain; g Hospital Clinico San Carlos, Idissc, Universidad Complutense, Madrid, Spain; h Všeobecná Fakultní Nemocnice v Praze, Prague, Czech Republic; i Centre Hospitalier Régional Universitaire de Besançon - Hôpital Jean Minjoz, University of Franche Comte SINERGIES, Besancon, France; j Berlin Institute of Health Center for Regenerative Therapies, Charité - Universitätsmedizin Berlin, Berlin, Germany; k Deutsches Herzzentrum der Charité, Department Cardiology (Virchow Klinikum), Charité - Universitätsmedizin Berlin, - German Centre for Cardiovascular Research Partner Site Berlin, Germany; l Center for Stroke Research Berlin, Charité - Universitätsmedizin Berlin, Berlin, Germany; m Laboratory of Cardiovascular Prevention, Istituto di Ricerche Farmacologiche Mario Negri IRCCS, Milan, Italy; n Mount Sinai Fuster Heart Hospital, The Mount Sinai Hospital, New York, USA

**Keywords:** Cost-effectiveness analysis, Cardiovascular prevention, CV-Polypill

## Abstract

**Background:**

The SECURE trial (NCT02596126) demonstrated the efficacy of the cardiovascular polypill (“CV-Polypill”–acetyl salicylic acid, atorvastatin and ramipril) in reducing the risk of recurrent major cardiovascular events compared with standard care when initiated within six months of a myocardial infarction. This analysis aimed to estimate the cost-effectiveness of the CV-Polypill from the Spanish healthcare perspective using SECURE trial data.

**Methods:**

A decision analytic Markov modelling approach was conducted to compare the CV-Polypill with standard care over a lifetime time horizon. Six parametric distributions were fitted to SECURE trial data on time to reinfarction, stroke or death (cardiovascular or non-cardiovascular). Cost and utility data were sourced from literature. Respective model outputs were discounted at 3%. The model captured direct medical costs associated with treatment acquisition and acute/ongoing cardiovascular events. Probabilistic sensitivity analyses (PSA) and scenario analyses were conducted.

**Findings:**

The CV-Polypill is dominant (improves health outcomes and reduces costs) in 84·8% of PSA iterations (848/1000 iterations), and cost effective in 89·3% of PSA iterations (893/1000 iterations) at a €30,000 threshold. Secondary prevention with the CV-Polypill reduces the recurrence of cardiovascular events and costs over the time horizon, from the Spanish healthcare perspective. A range of scenario analyses were conducted, demonstrating the robustness of the results when different inputs and assumptions were varied.

**Interpretation:**

The CV-Polypill is a dominant strategy in secondary cardiovascular prevention, compared with standard care, from the Spanish healthcare perspective. The CV-Polypill should be considered as a secondary prevention for Spanish patients, like those enrolled in SECURE, at hospital discharge.

**Funding:**

By 10.13039/501100018944Ferrer International.

**Research in context**

**Evidence before this study**

The SECURE study (NCT02596126) was a phase 3, randomised, controlled, outcomes trial designed to estimate the efficacy of the cardiovascular Polypill (“CV-Polypill” [acetyl salicylic acid, atorvastatin and ramipril]) compared with standard of care alone in the secondary prevention of cardiovascular disease. The study demonstrated that the CV-Polypill was associated with a lower risk of recurrent major adverse cardiovascular events and an increased probability of adherence, compared with standard of care. A pragmatic search was conducted to identify published literature to inform the cost effectiveness of the CV-Polypill. Initial evidence for the cost effectiveness of the CV-Polypill, from a Spanish cost-effectiveness model, was based on this improvement in adherence. Following this, another cost-effectiveness study was undertaken that modelled the improvement in cardiovascular risk factors with CV-Polypill compared with usual care. This further evidenced the cost effectiveness of the CV-Polypill.

**Added value of this study**

Post-hoc data analyses on the individual components of the SECURE composite primary outcome measure found that secondary prevention treatment with the CV-Polypill reduces the number of cardiovascular events and costs from the perspective of the Spanish healthcare system. As a consequence of this, when compared with standard care, the CV-Polypill represents a dominant strategy (increased benefits at lower cost). There was a high degree of certainty around these findings, with the probability of being cost-saving approximately 90%. While the cost-effectiveness of the CV-Polypill had been previously evaluated this is the first study to use survival analysis from study endpoints directly, as opposed to using general published risk equations.

**Implications of all the available evidence**

The CV-Polypill is both a clinically efficacious and cost-effective secondary prevention treatment that reduces the number of cardiovascular events and costs from a Spanish healthcare perspective, making it a strategy of choice upon hospital discharge.

## Introduction

Cardiovascular disease (CVD) is a major cause of morbidity and mortality in people globally, with the prevalence of myocardial infarction (MI) approaching three million people worldwide.^1^ Approximately 50%–75% of patients who experience MI will have a recurrent cardiovascular event within one to three years.^2^ CVD represents the leading cause of death in Spain (27·9% of deaths).^3^

Three of the drugs recommended by the European Society of Cardiology (ESC) to treat CVD include antiplatelets, statins and angiotensin-converting enzyme (ACE) inhibitors.^4^ However, an increase in medication complexity is associated with reduced adherence,^4^ with an estimated adherence of 50% and 66% for primary CVD prevention and secondary CVD prevention, respectively.^5^ Improved adherence to antiplatelet agents, statins and other CVD prevention medication can reduce the risk of further cardiovascular events, CVD-related morbidity and mortality, and healthcare costs due to rehospitalisation.^5^ A polypill strategy, combining several monocomponents into one tablet, improves treatment adherence,^6^ and a meta-analysis showed that patients receiving a polypill experienced a lower occurrence of cardiovascular events than control patients in primary prevention.^7^

The Spanish National Centre for Cardiovascular Research (CNIC) developed the CV-Polypill containing a combination of acetyl salicylic acid (ASA 100 mg), atorvastatin (20/40 mg), and ramipril (2·5/5/10 mg) which has been marketed and is used in several countries.

The CV-Polypill was found to reduce the risk of major adverse cardiovascular events (MACE) in secondary prevention patients in randomised prospective and non-interventional retrospective studies.^8^^,^^9^

Accordingly, the ESC included this polypill in their acute coronary syndrome (ACS) guideline to improve outcome and adherence after ACS (IIaB).^4^ Furthermore, the CV-Polypill is recommended for secondary prevention in hypertensive patients by the European Society of Hypertension (IIA)^10^ and was recently added to the 2023 Essential Medicines List published by the World Health Organisation for secondary CV prevention.^11^

### Phase III SECURE trial

The efficacy of the CV-Polypill was estimated in the Phase III Secondary Prevention of Cardiovascular Disease in the Elderly (SECURE), randomised, controlled, outcomes trial (NCT02596126), which examined the effects of polypill therapy on patients who had an MI within six months before enrolment.^8^ The trial was conducted in 113 centres across Europe, and all patients (N = 2499) were at least 65 years old with a history of one relevant risk factor (Supplementary Material Table S1). Patients were randomly assigned the CV-Polypill or standard care (monocomponents taken individually as per the ESC guidelines).^12^ Patients in the CV-Polypill arm could receive the Polypill AAR40 (a single pill containing ASA [100 mg], atorvastatin 40 mg and ramipril [2·5 mg, 5 mg or 10 mg]). The investigator could also reduce the atorvastatin dose to 20 mg based on the patient's history or blood test results. Further details regarding the possible CV-Polypill dosages are presented in the supplementary material (Table S2).

The primary outcome of the SECURE trial was a composite of cardiovascular death, non-fatal type one MI, non-fatal ischaemic stroke, or urgent revascularisation. The key secondary outcome was a composite of cardiovascular death, non-fatal MI, and non-fatal ischaemic stroke. In-person follow-ups occurred at 6, 12 and 24 months, with telephone follow-ups at 18, 36 and 48 months.

The CV-Polypill was associated with a significantly lower risk of MACE compared with standard care at a median follow-up duration of three years (primary outcome hazard ratio: 0·76 [95% CI: 0·60–0·96, p = 0·02]). The CV-Polypill was also associated with an increased probability of adherence compared with standard care at six and twenty-four months with risk ratios of 1·13 (95% CI: 1·06–1·20) and 1·17 (95% CI: 1·10–1·25), respectively. Further information on the SECURE trial is provided by Castellano et al.^8^

### Objective

To complement the clinical results from SECURE, a decision analytic economic model was developed to estimate the cost-effectiveness of the CV-Polypill in comparison with standard of care in secondary cardiovascular prevention from the perspective of the Spanish National Health System.

## Methods

### Patient population

The economic model was set up with the same mean baseline characteristics as patients within the SECURE trial. The starting age of the model population was assumed to be 76 years, and 69% of the population was male. A summary of further baseline characteristics of the hypothetical cohort simulated within the model is provided in the supplementary material (Table S3).

### Model structure

**Fig. 1:**
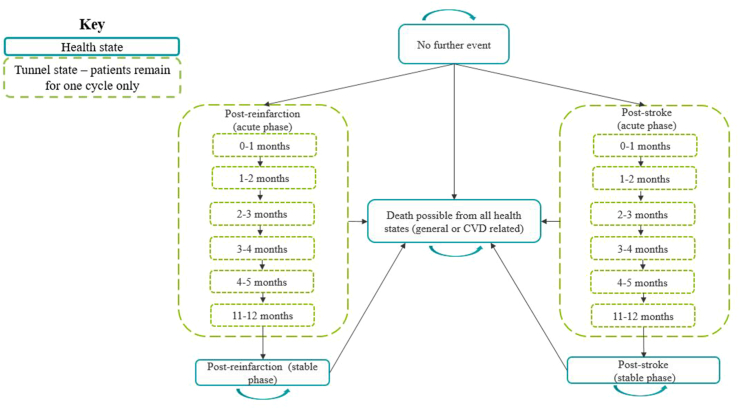
A Markov cohort model used to compare the costs and benefits of the CV-Polypill with those of standard care over a lifetime time horizon. Patients entered the model in the ‘no further event’ state, where they remained until they experienced reinfarction, stroke (disabling or non-disabling) or death (CVD or non-CVD related). Patients experiencing reinfarction or stroke transitioned through 12 monthly event-specific tunnel health states before reaching the ‘post-reinfarction’ or ‘post-stroke’ stable health states.

A Markov cohort model (Fig. 1) was developed to compare the costs and benefits of the CV-Polypill with those of standard care (monocomponents taken individually as per the ESC guidelines)^12^ over a lifetime time horizon. Costs and benefits were discounted at an annual rate of 3% in alignment with Spanish recommendations,^13^ and cost-effectiveness was assessed using a threshold of €30,000 per additional quality-adjusted life year (QALY) gained.^14^

A hypothetical cohort of patients that had experienced an MI in the previous six months entered the ‘no further event’ health state in the model. Patients remained in this state until they experienced reinfarction, stroke (disabling or non-disabling), or death (CVD or non-CVD related). Patients experiencing reinfarction or stroke transitioned through 12 monthly event-specific tunnel health states before reaching the ‘post-reinfarction’ or ‘post-stroke’ stable health states.

A monthly cycle was used to gradually model the changes in costs, mortality risks, and health-related quality of life (HRQoL) accrued by patients as they transitioned through the model. The targeted literature review (TLR) confirmed patients experienced worse outcomes (higher mortality and worse HRQoL) and utilised more resources in the first months following an event (Table 1)—tunnel states enabled these differences to be captured in the model.

**Table 1:** Key model input parameters.

Parameter	Model value	Source
**Monthly probability of death in the cycle directly after reinfarction or stroke (acute)**^a^	–	–
Reinfarction	4·84%	SECURE^8^: Data analysis
Stroke	2·22%	SECURE^8^: Data analysis
**Monthly probability of urgent revascularisation**	–	–
PCI	0·1%	SECURE^8^: Data analysis
**Event-specific SMRs**	–	–
Months 1–6 reinfarction	4·50	NICE^15^
Months 7+ reinfarction	3·00	NICE^15^
Months 1–6 stroke	4·73	NICE^15^
Months 7+ stroke	2·32	NICE^15^
**Health state utility values (applied per monthly cycle)**	–	–
Month 1 in the ‘no further event’ and ‘post-reinfarction’ health states	0·68 (AF: 0·75)	Lacey et al.^16^
Months 2–6 months in the ‘no further event’ and ‘post-reinfarction’ health states	0·78 (AF: 0·86)	NICE TA335/PLATO HECON study^17^
Months 7+ in the ‘no further event’ and ‘post-reinfarction’ health states	0·82 (AF: 0·91)	NICE TA335/PLATO HECON study^17^
‘Post-stroke’ (non-disabling)	0·81 (AF: 0·89)	NICE TA317^18^
‘Post-stroke’ (disabling)	0·45 (AF: 0·50)	NICE TA317^18^
**Disutilities (applied as one-off per even per cycle)**	–	–
PCI decrement	0·06	Samuel et al.^19^
**Monthly intervention and comparator treatment acquisition costs (per patient)**	–	–
CV-Polypill	€15·31	BotPlus^20^ (Accessed 24th May 2023, mandatory 7·5% discount applied to CV-Polypill^c^)
Standard care monocomponents	€17·67	BotPlus^20^ (Accessed 24th May 2023)
**Cardiovascular event costs (applied as one-off immediate event)**	–	–
Non-fatal reinfarction (APR-DRG-190)	€6288	Ministerio de Sanidad Subdirección General de Información Sanitaria. Registro de Actividad de Atención Especializada – RAE-CMBD (2021 costs)^21^
Fatal reinfarction (APR-DRG-190)	€12,026	Ministerio de Sanidad Subdirección General de Información Sanitaria. Registro de Actividad de Atención Especializada—RAE-CMBD (2021 costs)^21^
Non-fatal stroke (APR-DRG-047)	€5204	Ministerio de Sanidad Subdirección General de Información Sanitaria. Registro de Actividad de Atención Especializada—RAE-CMBD (2021 costs)^21^
Fatal stroke (APR-DRG-047)	€7473	Ministerio de Sanidad Subdirección General de Información Sanitaria. Registro de Actividad de Atención Especializada—RAE-CMBD (2021 costs)^21^
Non-cardiovascular death	€5670	National Institute of Statistics Spain^b^.^22^ Weighted average of costs per death in hospital setting (€5925). Cost per death not in hospital setting (€0). 2021 costs
Urgent revascularisation	€6036	Orly de Labry Lima, 2018.^23^ (Original value €5925)
**Follow-up costs**	–	–
GP visit	€92·59	BOE.^24^ núm. 180, de 29 de julio de 2013, páginas 55,225 a 55,251. (Original prices: GP visit: €90, Cardiologist: € 93).
Cardiologist outpatient visit	€95·36	
**Monthly ongoing health state costs**	–	–
‘No further event’	€31·33	Calculated by multiplying the unit cost of a GP visit and cardiologist outpatient visit by the health state resource use (as presented in Table S14, Supplementary Material)
‘Post-reinfarction’	€31·33	
‘Post-stroke’	€97·36	
**Societal costs**	–	–
Mean hourly pay in Spain	€14·42	Expatica^25^^,^^26^ Average annual salary in Spain: €29,994. Standard working week in Spain: 36·34 h.

**AF**, Adjustment factor; **BOE**, Boletín Oficial del Estado; **GP**, General Practitioner; **PCI**, Percutaneous Coronary Intervention; **SMR**, Standardised Mortality Ratio.

a Monthly probability of death, reinfarction and stroke in the ‘no further event’ health state are informed by the parametric distributions using data from the SECURE trial.

b 72,115 deaths. 266,235 (56·39%) were in the hospital setting. Data from 2020.

c The 7·5% discount is only applied to the CV-Polypill and is a mandatory pay back for medicines that are not included in a reference price system in Spain. This deduction is calculated by the Ministry of Health based on the prescriptions of CV-polypill and it is paid afterwards; it is not deducted at the time of dispensing, but as a payback.

**Table 2:** Economic evaluation results over a lifetime horizon (per patient).

	CV-Polypill	Standard care	Difference
Total cost discounted	€10,945	€11,537	−€592
Total QALYs discounted	6·70	6·62	0·08
Total life years discounted	9·29	9·19	0·10
Total life years undiscounted	11·40	11·26	0·14
Incremental cost-effectiveness ratio	–	–	Dominant
Net monetary benefit (per patient)	–	–	€2956

**CV**, Cardiovascular; **QALY**, Quality Adjusted Life Year.

**Table 3:** Predicted number of events over a lifetime horizon (per patient).

	CV-Polypill	Standard care	Difference
Number of reinfarctions	0·077	0·093	−0·016
Number of strokes	0·036	0·053	−0·017
Number of urgent revascularisations	0·090	0·089	0·001

**CV**, Cardiovascular.

The monthly probability of a patient within the ‘no further event’ health state experiencing a reinfarction, stroke, or cardiovascular death was informed by parametric survival analysis using individual patient-level data (IPD) from the SECURE trial (as described in Tables S4 to S9 of the supplementary material). No treatment waning effect was applied in the base case analysis due to the lack of long-term data to inform the likelihood of this. However, scenario analyses were conducted to investigate the impact of an instant or gradual treatment waning effect following the four-year follow-up period of the SECURE trial. The survival analysis was adjusted to ensure that the overall rate of death could never be lower than the general population mortality rates in Spain (adjusted for age and sex distribution).^27^

It was assumed that patients could not experience more than one reinfarction or stroke due to the lack of information available in the literature to model the frequency and outcomes of downstream events; this assumption aligns with previous models published in this area (including a model used to inform UK guidelines)^28^ and was considered reasonable by clinical experts at an advisory board. Therefore, patients remained in one of the two stable health states after a recurrent event until death. Patients could also die whilst in any other health state. Additionally, it was assumed that patients would remain on treatment following the recurrence of either a stroke or an MI. This impacted treatment costs only, and no treatment effects were applied once patients left the no further event state. This was a conservative assumption made due to a lack of data to inform the treatment effect after the completion of the clinical trial. The impact of the CV-Polypill on non-CVD outcomes was not considered in the model because data specifically on CV outcomes due to diabetes mellitus were not collected in the SECURE trial and the inclusion of such outcomes would have been associated with considerable uncertainty. However, the CV-Polypill did not increase all-cause mortality (HR 0·97 (95% CI: 0·75–1·25)) compared with standard of care in the SECURE study.

Patients were at risk of requiring urgent revascularisation (percutaneous coronary intervention [PCI]) whilst in any of the health states. There was no limit on the number of urgent revascularisations patients could experience throughout the model time horizon (as validated by clinical experts), and the probability of requiring a PCI was the same in all health states. A one-off utility decrement and cost were applied to the proportion of patients who experienced urgent revascularisation in each cycle (as presented in Table 1).

The model conceptualisation and key assumptions were validated by a panel of four cardiologists (including three SECURE trial investigators) and two health economic experts.

### Additional model input parameters

The model input parameters are presented in Table 1. The model inputs were obtained from the SECURE trial data where possible,^8^ and a TLR was conducted to fill any evidence gaps.

#### ‘Post-event’ mortality

All inputs informing mortality probabilities after reinfarction or stroke (i.e. the ‘post-event’ health states) were informed by the TLR given that the number of deaths after these events in the SECURE trial was deemed insufficient to perform a robust analysis. These probabilities were not treatment dependent. A higher probability of death was applied to patients in the first cycle after reinfarction or stroke to account for the higher risk of death in the immediate month following an event (i.e. whilst in the first tunnel health state) (Table 1). The increased ongoing risk of death post-event (i.e. in the second tunnel state onwards and the stable health states), compared with the general population, was captured through the application of standardised mortality ratios (SMRs) to Spanish-specific general population mortality rates.^27^

#### Utilities

HRQoL data were collected in the SECURE trial (at baseline and two years), through the EQ-5D-3L questionnaire.^29^ Due to this data collection schedule it was not possible to inform the HRQoL associated with the post-event health states using this source. Further, the trial baseline values (0·858 and 0·857) indicated that participants had higher HRQoL values than the age and sex-adjusted general Spanish population calculated using values from Szende et al.^30^ Experts at a clinical advisory board agreed that the HRQoL of the SECURE population should have been lower than the general population and, therefore, utility values identified from the TLR were used to inform the base case analysis.

The results of a scenario analysis using the utility data from the SECURE trial have also been presented in Table 4. A summary of the utility values used in each health state is presented in Table 1.

**Table 4:** Scenario Analyses (per patient, cost-effectiveness threshold: €30,000).

	Δ Cost	Δ QALY	ICER	INMB
Base case	−€592	0·08	Dominant	€2956
Second-best fitting distributions for parametric survival curves according to AIC/BIC (log-logistic for all)	−€596	0·09	Dominant	€3424
Gradual treatment waning effect following SECURE trial (four to ten years)	−€486	0·07	Dominant	€2452
Instant treatment waning effect following SECURE trial (four years)	−€425	0·05	Dominant	€2029
50% of patients who remain on treatment post-stroke and reinfarction	−€571	0·08	Dominant	€2935
Societal perspective	−€1121	0·08	Dominant	€3486
SECURE time horizon	−€297	0·01	Dominant	€510
‘No further event’ health state utility value informed by the SECURE trial	−€592	0·10	Dominant	€3469
No treatment effect on all efficacy parameters	−€260	0·00	Cost saving	€260
Equivalent dosages across arms	−€282	0·08	Dominant	€2647

**AIC**, Akaike Information Criterion; **BIC**, Bayesian information criterion; **ICER**, Incremental Cost-Effectiveness Ratio; **INMB**, Incremental Net Monetary Benefit; **QALY**, Quality Adjusted Life Year.

Separate utility values were applied depending on whether patients experienced a disabling or non-disabling stroke. The nature of each stroke was not collected in the SECURE trial and, hence, the proportion of disabling strokes (46%) was obtained from the PLATO HECON study that was based on patients with ACS^31^—this study was considered robust due to the large sample size (over 18,000 patients with ACS were recruited). Patients in the ‘no-event’ health state were assumed to have experienced an MI within a median of eight days of entering the model.^8^ Therefore, the utility values were assumed to be equivalent between the ‘no event’ and ‘post-reinfarction’ health states (i.e. a lower utility shortly after the MI [including the initial MI upon model entry] which increases once stable).

The health state utilities values were divided by general population utility values (reported by Szende)^30^ to estimate an adjustment factor which was multiplied by general population utilities to determine age-specific utilities for each health state.^30^ A further one-month disutility was applied to the proportion of patients experiencing revascularisation for one month within each cycle (this duration was validated by clinicians at the advisory board).

#### Costs

Treatment acquisition costs, cardiovascular event costs (hospital tariff codes), and health state-specific healthcare resource use unit costs were included in the model. The frequency of each resource use was multiplied by the unit cost, and costs were aggregated to produce the total costs of treatment with the CV-Polypill and standard care. Costs were sourced from recognised Spanish resources wherever possible, and all unit costs were sourced in the 2023 cost year. Where no 2023 data were available, historical values were inflated to 2023 equivalents using appropriate inflation indices.^31^

Patients were assumed to consistently receive treatment with the CV-Polypill or standard care throughout the time horizon, regardless of health state (although treatment discontinuation assumptions were varied within a scenario analysis). The cost applied to the CV-Polypill was calculated using a weighted average based on the proportion of each of the formulations prescribed within the SECURE trial and the weighted average of each dosage (Table S10, Supplementary Material). The standard care costs were calculated as a weighted average based on the proportion of each of the individual monocomponents used within the SECURE trial and the relevant unit costs. As patients assigned to the standard care arm of the trial were not restricted to being prescribed the individual monocomponents within the CV-Polypill, other individual ACE inhibitors and statins were included in this weighted average. Further detail on these calculations is presented in Tables S11–S13 (Supplementary Material).

A one-time in-hospital tariff cost was applied to patients who experienced reinfarction or stroke to account for the initial costs associated with either event. It was assumed that patients would also require ongoing visits with cardiologists and primary care doctors thereafter. The unit costs associated with these are outlined in Table 1, and the resource use estimates are presented in Table S14 (Supplementary Material). The same healthcare professional costs were applied to patients within both the ‘no further event’ and ‘stable reinfarction’ health states as the ongoing healthcare resource use was deemed unlikely to differ following an additional event. A one-time cost was also applied to patients who experienced fatal reinfarction or fatal stroke (as determined by time to cardiovascular death within the SECURE trial [Table 1]).

### Sensitivity analysis

Probabilistic sensitivity analyses (PSA) were undertaken to investigate second-order uncertainty. All model inputs were sampled 1000 times using underlying parameter distributions. To generate the input values for each iteration, distributions were fitted to uncertain parameters within the model. For probabilities and utilities, beta distributions were used, while cost parameters were fitted with gamma distributions. Uncertainty around estimates provided by the regression equations was incorporated by utilising Cholesky decompositions.

### Scenario analyses

A series of deterministic scenario analyses (described in supplementary material) were performed to assess the robustness of the results when different inputs or assumptions were varied.

### Role of the funding source

Ferrer has contributed to the study design, data analysis interpretation and writing of the manuscript, in collaboration with the academic authors. This entailed revision of literature for the model conceptualisation, data acquisition and revision of the statistical analysis plan, model and manuscript. However, no employee of Ferrer has been directly involved in the critical review of the intellectual content and, therefore, has not approved the final text.

## Results

### Base case analysis

The CV-Polypill is associated with hazard ratios of 0·66 (95% CI: 0·36–1·22) and 0·77 (95% CI: 0·50–1·19) when compared with standard care for time to stroke and CVD death, respectively. Furthermore, the estimated log time ratio to reinfarction was 0·42 (95% CI: −0·27 to 1·11).

Results over a lifetime time horizon show that the CV-Polypill is dominant (less costly and more effective) when compared with standard care (Table 2). The CV-Polypill remains dominant up to a unit cost of €20·61 (€0·68 per day).

The CV-Polypill is associated with fewer reinfarctions and strokes compared with standard of care (Table 3). A detailed cost breakdown is presented in Table S16 (Supplementary Material). An accrued health state occupancy graph is presented in Figure S9 (Supplementary Material).

### Sensitivity analysis

**Fig. 2:**
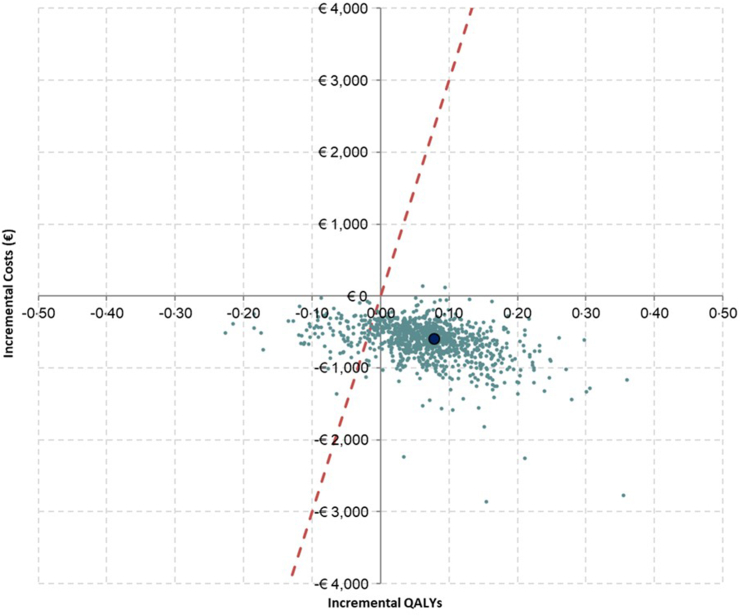
Most iterations fell into the southeast quadrant of the plane below the threshold of €30,000 per additional QALY gained.

The results of the PSA are aligned with the base case results, with the CV-Polypill being less costly and generating more QALYs than standard care. A cost-effectiveness plane displaying the PSA results is presented in Fig. 2. Most iterations fell in the southeast quadrant of the plane. The results show that the probabilities of the CV-Polypill being dominant and cost effective are 84·8% and 89·3%, respectively.

### Scenario analyses

The results from each of these analyses are presented in Table 4. The CV-Polypill remains dominant in all bar one, with it being cost saving in the outstanding scenario. Incremental net monetary benefit was positive in all scenarios explored.

## Discussion

The results of this cost-effectiveness analysis suggest that the CV-Polypill is a dominant strategy for the secondary prevention of cardiovascular events and offers patients more QALYs at less cost than standard care. The results from the analyses of SECURE trial data show that the CV-Polypill reduces MACE. CV-Polypill was cost-effective in 89·3% of all PSA simulations (using a threshold of €30,000 per QALY gain) and dominant in 84·8% of simulations. Scenario analyses showed that the CV-Polypill remains dominant or cost-saving in all scenarios. As such, the CV-Polypill should be considered the therapeutic strategy of choice for the prevention of secondary cardiovascular events because it would contribute to the sustainability of the Spanish healthcare system and help alleviate resource constraints.

This is the first analysis to date to investigate the cost-effectiveness of the CV-Polypill in comparison with standard care by modelling cardiovascular outcomes directly from a clinical trial. Prior analyses were based on risk factor change and/or adherence improvement.^32^, ^33^, ^34^, ^35^, ^36^ The hypothetical cohort simulated through the economic model was designed to have the same baseline characteristics as those in the SECURE trial. The results showed that the CV-Polypill prevents 40 reinfarctions and 42 strokes over a lifetime time horizon based on the SECURE trial patient cohort (N = 2466).

As is the case in many other European countries, CVD is a primary cause of death in the Spanish population (27·9% of deaths).^3^ Therefore, this presents a valid public health concern. Poor medication adherence contributes to high rates of repeat events following an acute MI.^6^ The FOCUS study (NCT00567307) determined that CV-Polypill improved medication adherence over a nine-month follow-up period compared with standard care (50·8% versus 41%, p = 0·019, intention-to-treat population).^6^

The results of the retrospective non-interventional NEPTUNO study with four propensity score-matched cohorts, supported the results of the SECURE trial in a real-life setting of patients in secondary prevention. This study, which analysed a total of 6456 patients, showed that after two years, the risk of recurrent MACE was 22%, 25% and 27% higher with monocomponents, equipotent and other therapies compared with the CV-Polypill.^9^

The cost-effectiveness of the polypill strategy has been investigated in other secondary prevention settings, and the results from this cost-effectiveness analysis align with previously published work,^32^, ^33^, ^34^, ^35^, ^36^ two of which found the polypill approach to be a cost-effective strategy from the perspective of the Spanish healthcare system.^33^^,^^35^ In one of these studies by Barrios et al., the polypill had the same price as the generic monocomponents. Becerra et al. and Aguiar et al. both concluded that polypill was cost-effective in a UK and Portuguese setting, respectively.^32^, ^33^, ^34^ Aguiar et al.^32^ stated that the polypill had a slightly higher incremental cost than the generic monocomponents and Becerra et al.^34^ stated that the polypill was more expensive than generic monocomponents. These previously published cost-effectiveness analyses were based on improvements in adherence^33^^,^^34^ or risk factor control^35^^,^^36^ between arms. The benefit of the present study is that this is the first study to use survival analysis from study endpoints directly. The present model estimates that the CV-Polypill is associated with discounted QALY and undiscounted life year gains of 0·08 and 0·10, respectively, per person. These gains are comparable to those observed in the aforementioned models which report an average QALY and life-year gain of 0·03 per person.

A strength of this analysis is that the composite secondary endpoint from the SECURE trial was incorporated into the model to inform the time to reinfarction, stroke and death within the CV-Polypill and standard care arms.

Due to the follow-up period within the SECURE trial, it was necessary to extrapolate the observed time to reinfarction or stroke information using parametric functions. This is a standard approach in decision analytic modelling and ensures that the lifetime costs and benefits of each treatment are estimated, and hence a fair evaluation of cost-effectiveness is undertaken. Furthermore, the Kaplan–Meier data used to inform these extrapolations were relatively immature. While the choice of extrapolation was validated by clinical experts, such extrapolations are associated with uncertainty. Although it is also unclear whether a treatment waning effect will be experienced by patients taking the CV-Polypill, it remained dominant in all scenarios where this was varied.

It was necessary to make assumptions when developing the model—some favoured the CV-Polypill and others favoured standard care. In particular, it was assumed that patients could only experience one additional event (reinfarction or stroke), and they could not experience repeat cardiovascular events. This assumption was necessary to reduce the complexity of the model structure, which was important due to a paucity of data available to accurately estimate downstream cardiovascular events. This is a conservative assumption that underestimates the benefits of the CV-Polypill. This assumption was also made in previously published cardiovascular models and validated by clinical experts within an advisory board.^15^^,^^34^ It was assumed that follow-up care costs are similar in both arms due to an absence of alternative data—this may favour the CV-Polypill.

The impact of the CV-Polypill on non-CVD outcomes was not considered in the model. Previous studies have demonstrated that statins may cause an increase in new diagnoses of type 2 diabetes mellitus.^36^ While there is a modest increased risk of diabetes from the use of statins, the most significant sequelae from any increased risk of diabetes are the risk of future CVD events and mortality. The SECURE primary endpoint included CVD mortality, non-fatal MI and non-fatal stroke. Therefore, the mortality hazard ratio between the two arms reflected the reductions in these events despite the increased risk from potential diabetes and was incorporated in the model estimates intrinsically. The CV-Polypill was not associated with an increased dose of statins in the SECURE trial and, therefore, there will likely not be an increased risk of type 2 diabetes mellitus associated with the CV-Polypill. Furthermore, the inclusion of such outcomes would have been associated with considerable uncertainty due to a lack of data availability.

Another potential limitation of this study is the approach used to collect HRQoL data in the SECURE trial. It was not possible to calculate utility decrements associated with experiencing either reinfarction or stroke because these data were collected at only baseline and two years. Furthermore, the results indicated that participants had a higher HRQoL than the general population which was not considered to be realistic. Therefore, the clinical experts recommended the use of utility values, informed from the literature. Furthermore, while additional resources (such as diagnostic tests) will be incurred by patients following stroke or reinfarction, these were not included in the model because they were viewed as being unlikely to be key drivers of the cost-effectiveness results and were the same across both arms of the model.

According to the ESC guidelines, lipid-lowering treatment may be required for a subset of patients to lower low-density lipoprotein-cholesterol levels.^4^ Thus, the additional costs of lipid-lowering treatment, and the likely reduction of clinical events, may alter the results of the cost-effectiveness analysis.

Treatment adherence is important in cardiovascular prevention. Previous literature and the sensitivity analysis in the current analysis suggest a polypill strategy provides a possible advantage in terms of adherence. In future, real-world data could be used to update the analysis with information associated with the adherence to CV-Polypill outside of the trial setting.

Finally, the results of our analysis may not be readily generalisable to other European countries due to pricing variability. Specifically, whilst the CV-Polypill is cheaper than generics in Spain, the price of the CV-Polypill may be higher than that of the monocomponents in other countries.

### Conclusion

The CV-Polypill (ASA, atorvastatin and ramipril) is a dominant strategy when compared with standard care administered concomitantly/individually from a Spanish healthcare perspective. The CV-Polypill is cost-saving compared with standard care over a lifetime time horizon, as well as in most of the sensitivity and subgroup analyses. The results of this work suggest that the CV-Polypill can reduce the incidence of cardiovascular events and the overall costs associated with the treatment of MACEs, making it a valuable strategy that should be considered as core therapy upon hospital discharge in Spanish patients meeting the SECURE inclusion criteria.

## Contributors

A. Dymond, A. Looby and S. Mealing developed the model and wrote the manuscript. R. Owen led the statistical analysis. T. Gaziano, J.M. Castellano, A. Dymond, A. Looby, S. Mealing, R. Owen, S. Pocock, J.R. González-Juanatey, A. Cordero, A. Fernández-Ortiz, A. Linhart, F. Schiele, W. Doehner, L.O. Fernández, and V. Fuster contributed to the model conceptualisation, interpretation of the results, and reviewed the final manuscript.

## Data sharing statement

All data used to inform the model are available in the manuscript.

## Declaration of interests

This cost-effectiveness analysis was funded by Ferrer International. T. Gaziano, JM. Castellano, A. Cordero and JR. González-Juanatey participated on an advisory board supported by Ferrer. A. Dymond, A. Looby and S. Mealing are employees of York Health Economics Consortium and were commissioned by Ferrer to provide consultancy, conceptualise and build the model, and draft the manuscript. The funders contributed to the study design, interpretation of data and writing of the manuscript. In the last 36 months, T. Gaziano has received a grant from Novartis and NIH, and also received consulting fees from Novartis, Astra-Zeneca, Multply Labs, and Amgen. In the last 36 months, A. Linhart has received a research grant from Sanofi; consultaing fees from Novo Nordisk and Sanofi; speaker fees from Servier and Chiesi; participated in an advisory board for Novo Nordisk; and received laboratory material from Takeda. In the last 36 months, W. Doehner received research support from Vifor (to institution), Boehringer Ingelheim (to institution) and the EU commission; consulting fees from Aimediq, Bayer, Boehringer Ingelheim, Boston Scientific, Medtronic, Vifor Pharma, Cardiomatics, and Icon Clinical Research Ltd; travel support from Pharmacosmos; participated on an independent data monitoring board for Boehringer Ingelheim and IQVIA, and an adjudication board for IQVIA; is an unpaid councillor of the ESC board, an unpaid Nuclus members on the ESC Council on Stroke, and is an unpaid board member for the Sociert of cachexia and wasting disorders. A. Cordero received online meeting support from Ferrer relating to the present manuscript and, in the last 36 months, received consulting fees from AstraZeneca, Ferrer, Sanofi, AMGEN, Novartis, Lilly, Novo Nordisk, Daiichy Sankio and Amarin; payment or honoraria for lectures, presentations, speakers bureaus, manuscript writing or educational events from AstraZeneca, AMGEN, Bristol-Myers Squibb, Ferrer, Boehringer Ingelheim, MSD, Daiichy Sankio, Novartis, Novo Nordisk, Sanofi and Amarin; payment for expert testimony from Ferrer, AMGEN, and MSD; Support for attending meetings and/or travel from Ferrer, AMGEN, MSD, and Novo Nordisk.
