# Supplementary material for: Cost-effectiveness of the CV-polypill strategy versus standard care for secondary cardiovascular prevention in Spain: an analysis based on the SECURE trial
Source: Lancet Reg Health Eur. 2025 Jun 27;55:101348. doi: 10.1016/j.lanepe.2025.101348 (PMC12269512; doi:10.1016/j.lanepe.2025.101348)
Supplement: Supplementary Material [file mmc1.pdf]

Supplementary Materials:

Contents:

Methods
Patient population and characteristics page 1
Statistical analysis of individual patient data from the SECURE trial page 2
Additional model input parameters page 8
Scenario analyses page 10
Results
Additional base case analysis results page 14

Methods

Patient population and characteristics

Table S1: Eligibility Criteria for SECURE trial [1]

**Inclusion Criteria**
Giving consent after information
Patients diagnosed with a type 1 myocardial infarction within the previous 6 months
Subjects must be ≥ 65 years old presenting with one of the following additional conditions<br>1. Documented diabetes mellitus or previous treatment with oral hypoglycemic drugs or insulin<br>2. Mild to moderate renal dysfunction: creatinine clearance 60-30mL/min/1·73m^2<br>3. Prior to myocardial infarction: defined as an acute myocardial infarction occurring before the index event documented in a medical report<br>4. Prior coronary revascularization: coronary artery bypass grafting or percutaneous coronary intervention<br>5. Prior stroke: history of a documented stroke, defined as an acute episode of focal cerebral, spinal, or retinal dysfunction caused by infarction of central nervous system tissue, not resulting in death<br>6. Age ≥ 75 years

Table S2: Polypill combinations

**ASA/Atorvastatin/Ramipril**
100/20/2·5 mg
100/20/5 mg
100/20/10 mg
100/40/2·5 mg
100/40/5 mg
100/40/10 mg

Table S3: Population characteristics

	**Input**	**Source**
Starting age of population	76	SECURE [1]
Percentage male	69·0%	
Proportion with diabetes	42·6%	
Proportion of smokers	14·4%	
Proportion of previous smokers	39·9%	
Proportion with hypertension	77·9%	
Proportion with hyperlipidaemia	58·5%	
Proportion non-disabling strokes	54·0%	NICE [2]
Proportion disabling strokes	46·0%	
Proportion of mild myocardial infarctions	29·7%	Rabier [3]
Proportion of severe myocardial infarctions	70·3%	

Statistical analysis of individual patient level data from the SECURE trial [1]

Individual patient data from 2,466 patients who were enrolled in the SECURE trial were used to derive prognostic equations to inform input parameters for the cost-effectiveness model. A complete case analysis (N=2,305) was conducted, and right censoring was applied to all time-to-event analyses. Where there was less than ten percent available of the information 'missing' values were not imputed and the data was assumed to be missing completely at random (MCAR).[4] Statistical analyses were performed in STATA version 17·0.[5] The survival analysis methods were aligned with best practice guidance.[6]

A range of parametric survival models (exponential, Weibull, Gompertz, log-normal and log-logistic) were used to estimate time to reinfarction, stroke, cardiovascular disease (CVD) death, and non-CVD death from the point of randomisation. The models were based on the following competing risks:

- Time to myocardial infarction: Conditional on not having a stroke or dying from any cause.
- Time to stroke: Conditional on not having a reinfarction or dying from any cause.
- Time to cardiovascular death: Conditional on not having a reinfarction or stroke.

The proportional hazards (PH) assumption was tested using a Cox regression model and was met for all survival curves. Therefore, one model was applied to both treatment arms (with a treatment covariate applied to the treatment arm). The baseline covariates included CV-Polypill, age, sex, diabetes, smoking history, hypertension, and hyperlipidaemia. The coefficients associated with each analysis are presented in Tables S4 to S8.

For each parametric survival model, the Akaike information criterion (AIC) and Bayesian information criterion (BIC) goodness of fit statistics were extracted, and the clinical plausibility was reviewed. The AIC/BIC estimates (Table S9) indicated that the log-normal, Weibull and exponential curves were the best fitting for time to reinfarction, stroke and CVD death, respectively (the curves used in the base case are displayed in Figures S1 to S4). The Gompertz curve was best fitting for time to non-CVD death when the AIC/BIC was considered alone. However, the Weibull curve was considered best fitting as judged by clinical plausibility; therefore, this was used in the base case analysis.

Table S4: Time to reinfarction coefficients

	Exponential	Weibull	Gompertz	Log-normal	Log-logistic
Rate	-11·793***		-10·837***		
Shape		0·641***	-0·001***		1·536
Scale		-·0881***			14·139***
Meanlog				16·569***	
Sdlog				3·730	
Mu					
Sigma					
Q					
Polypill	-0·304	-0·305	-0·304	0·421	0·473
Age	0·017***	0·014	0·013	-0·032	-0·023
Sex	0·192	0·202	0·203	-0·465	-0·329
Diabetes	0·472*	0·445*	0·438*	-0·807*	-0·707*
Smoker	-0·354	-0·375	-0·381	0·639	0·584
Previous smoker	0·139	0·135	0·133	-0·197	-0·215
Hypertension	-0·014	-0·011	-0·009	0·028	0·019
Hyperlipidaemia	0·435	0·415	0·409	-0·670	-0·653

* P<0·05; ** P<0·01 ***P<0·001

Table S5: Time to stroke coefficients

	Exponential	Weibull	Gompertz	Log-normal	Log-logistic
Rate	-12·783***		-12·417***		
Shape		0·669***	0·000		1·488
Scale		-10·299			15·393***
Meanlog				18·946	
Sdlog				4·214***	
Mu					
Sigma					
Q					
Polypill	-0·409	-0·408	-0·408	0·783	0·616
Age	0·027	0·025	0·025	-0·048	-0·037
Sex	0·111	0·123	0·117	-0·176	-0·181
Diabetes	0·142	0·116	0·127	-0·354	-0·181
Smoker	-0·551	-0·570	-0·561	0·822	0·849
Previous smoker	-0·418	-0·422	-0·421	0·691	0·632
Hypertension	0·249	0·249	0·250	-0·187	-0·368
Hyperlipidaemia	-0·048	-0·065	-0·059	-0·006	0·092

* P<0·05; ** P<0·01 ***P<0·001

Table S6: Time to CVD death coefficients

	Exponential	Weibull	Gompertz	Log-normal	Log-logistic
Rate	-18·277		-18·390***		
Shape		0·906	0·000		1·089
Scale		-17·550***			19·410***
Meanlog				21·626***	
Sdlog				2·830	
Mu					
Sigma					
Q					
Polypill	-0·254	-0·254	-0·254	0·390	0·289
Age	0·090***	0·089***	0·091***	-0·108***	-0·100***
Sex	0·198	0·199	0·199	-0·147	-0·201
Diabetes	0·506*	0·499*	0·510*	-0·735*	-0·571*
Smoker	0·382	0·378	0·384	-0·463	-0·423
Previous smoker	0·606*	0·605*	0·607*	-0·982**	-0·700*
Hypertension	0·335	0·336	0·334	-0·409	-0·379
Hyperlipidaemia	0·525*	0·519*	0·528*	-0·551	-0·564*

* P<0·05; ** P<0·01 ***P<0·001

Table S7: Time to non-CVD death coefficients

	Exponential	Weibull	Gompertz	Log-normal	Log-logistic
Rate	-16·537***		-17·218***		
Shape		1·192	0·001**		0·824
Scale		-17·981***			15·149***
Meanlog				17·046***	
Sdlog				2·105	
Mu					
Sigma					
Q					
Polypill	0·458*	0·459*	0·460*	-0·508*	-0·402*
Age	0·075***	0·076***	0·078***	-0·077***	-0·066***
Sex	0·069	0·065	0·063	-0·052	-0·055
Diabetes	0·439*	0·455*	0·470*	-0·411*	-0·390*
Smoker	0·786**	0·793**	0·798**	-0·887**	-0·703**
Previous smoker	0·208	0·211	0·215	-0·169	-0·181
Hypertension	0·188	0·186	0·183	0·000	-0·147
Hyperlipidaemia	-0·058	-0·050	-0·039	-0·025	0·039

* P<0·05; ** P<0·01 ***P<0·001

Table S8: Rate of urgent revascularisation: Does not change across health states

Variable	Deterministic
Constant	-3·942*
Age	-0·013
Sex	0·614
Diabetes	0·142
Smoker	-0·477
Previous smoker	0·014
Hypertension	0·373
Hyperlipidaemia	0·534

* P<0·05; ** P<0·01 ***P<0·001

Table S9: AIC/BIC values

Distribution	Time to reinfarction		Time to stroke		Time to CVD-death		Time to non-CVD death	
	AIC	BIC	AIC	BIC	AIC	BIC	AIC	BIC
Exponential	1112·70	1164·39	563·50	615·19	882·98	934·67	1004·69	1056·37
Weibull	1089·62	1147·04	556·60	614·02	883·99	941·42	1003·02	1060·45
Gompertz	1092·63	1150·06	564·18	621·60	884·80	942·23	997·72	1055·14
Log-normal	1087·04	1144·47	558·71	616·14	890·71	948·14	1013·01	1070·44
Log-logistic	1089·37	1146·80	556·69	614·12	884·35	941·78	1003·39	1060·82

AIC, Akaike Information Criterion; BIC, Bayesian information criterion

Figure S1: Time to reinfarction - Log-normal

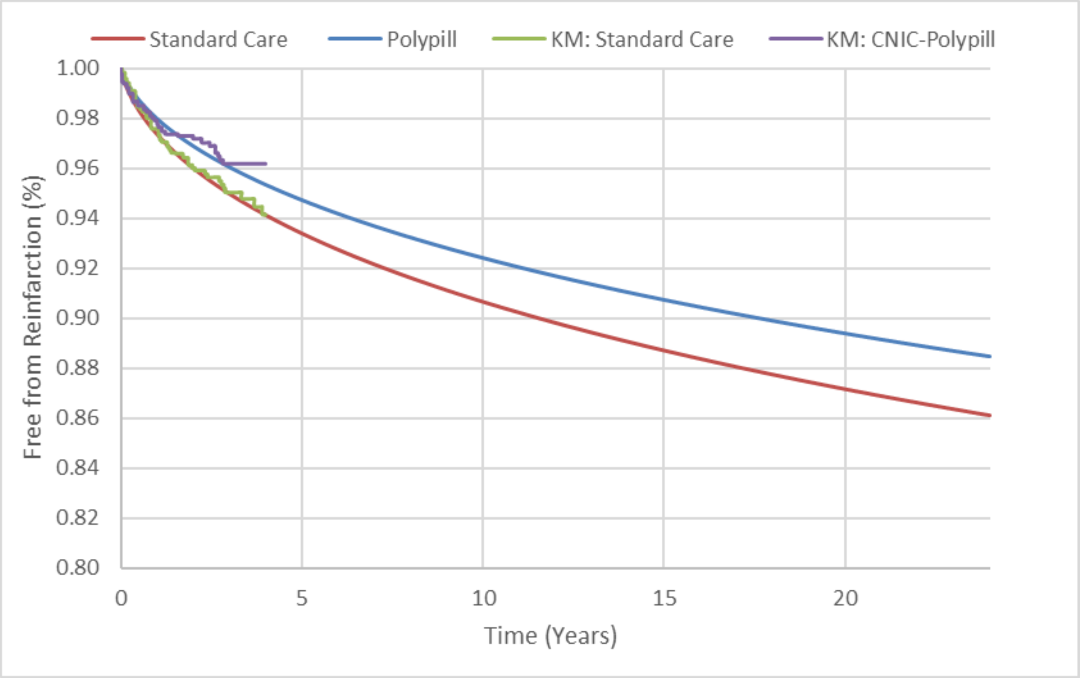

Figure S2: Time to stroke – Weibull

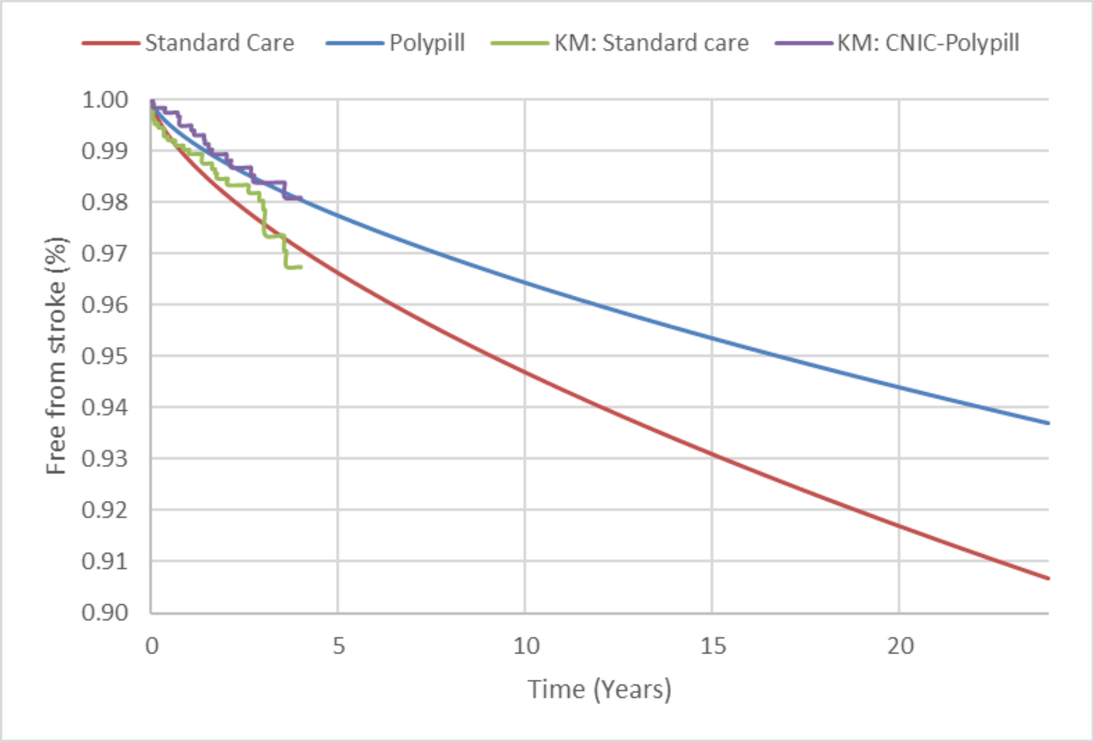

Figure S3: Time to CVD death – Exponential

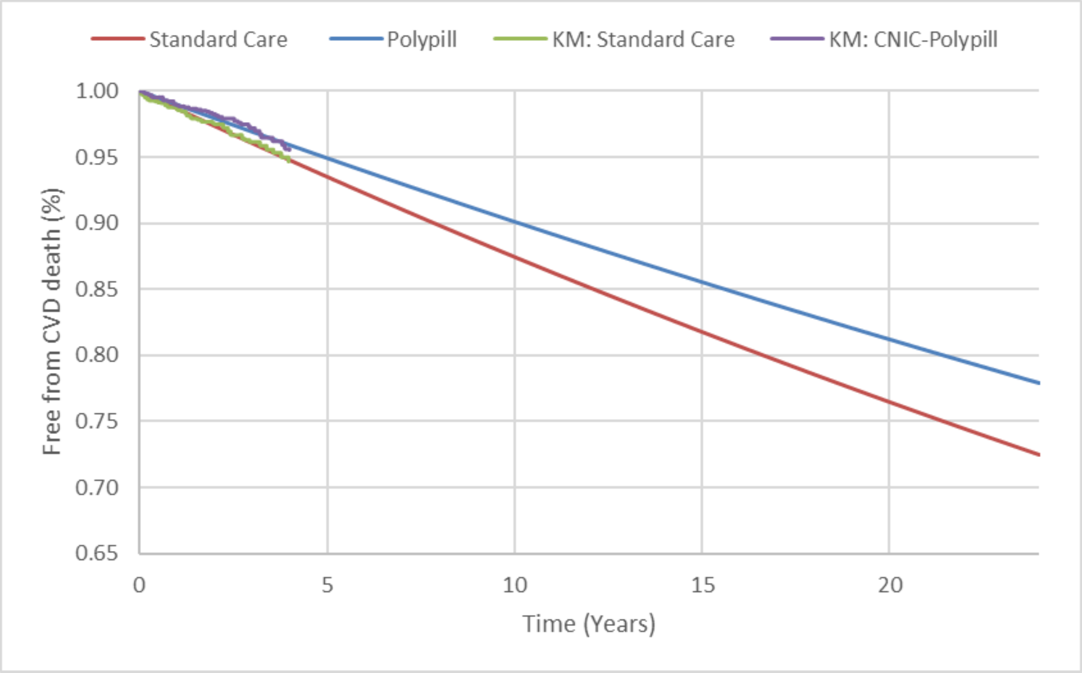

Figure S4: Time to non-CVD death – Weibull

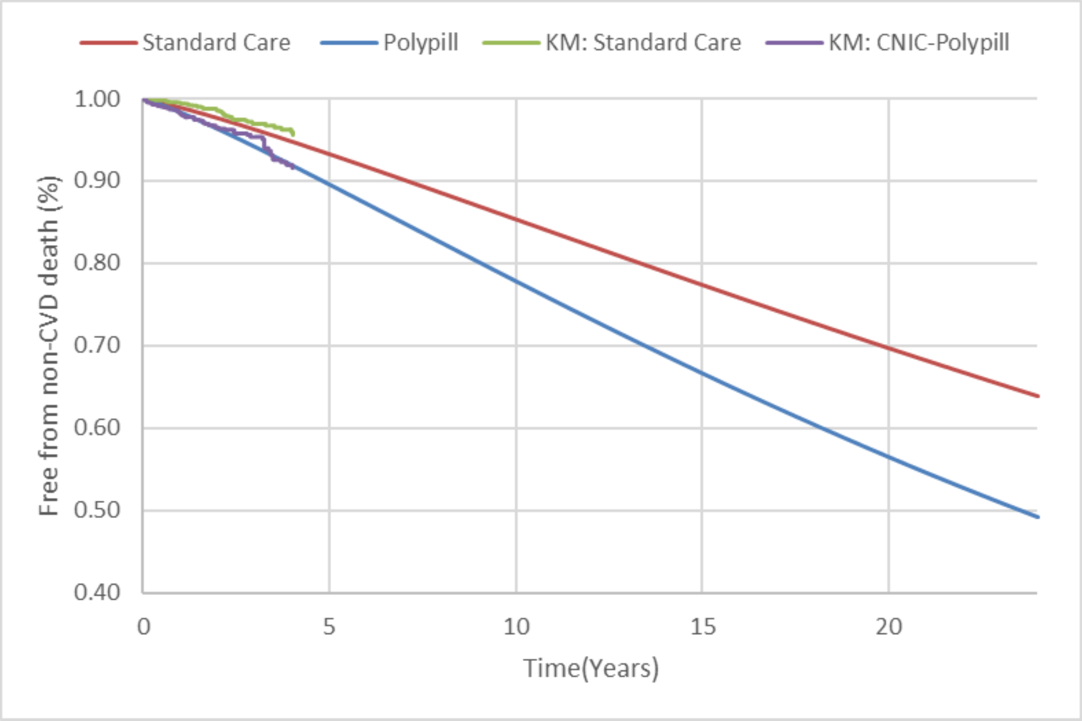

Additional model input parameters

Table S10: Polypill treatment unit costs and combination usage (assumed 30.42 tablets per month)

	Usage	Unit cost	Cost (incl. discount)	Pack size	Cost per tablet	Total cost per cycle	Source
100/20/2·5 mg	4·0%	€8·37	€7·74	28	€0·28	€8·41	SECURE.[1] BotPlus,[7] accessed 24th May 2023.
100/20/5 mg	2·5%	€9·87	€9·13	28	€0·33	€9·92	
100/20/10 mg	1·8%	€12·97	€12·00	28	€0·43	€13·03	
100/40/2·5 mg	40·9%	€14·27	€13·20	28	€0·47	€14·34	
100/40/5 mg	32·3%	€15·77	€14·59	28	€0·52	€15·85	
100/40/10 mg	18·5%	€18·87	€17·45	28	€0·62	€18·96	
**Weighted average**						€15·31	

Note: Aspirin/atorvastatin/ramipril

Table S11: Statin treatment unit costs and usage

	Usage	Mg per day	Unit Cost (per pack)	Pack size	Cost per mg	Mg per cycle	Total cycle cost	Source
High intensity								SECURE.[1] BotPlus,[7] accessed 24th May 2023·
Atorvastatin 40mg	42·3%	40	€10·48	28	€0·01	1,217	€11·38	
Atorvastatin 80mg	36·3%	80	€20·96	28	€0·01	2,433	€22·77	
Rosuvastatin 20mg	4·1%	20	€12·63	28	€0·02	608	€13·72	
Moderate intensity								
Atorvastatin 10-20mg	8·1%	15	€4·67	28	€0·01	456	€5·07	
Rosuvastatin 5-10mg	1·2%	8	€4·74	28	€0·02	228	€5·14	
Simvastatin 20-40mg	3·8%	30	€1·20	28	€0·00	913	€1·30	
Pravastatin 40mg	0·2%	40	€10·44	28	€0·01	1,217	€11·34	
Lovastatin 40mg	0·2%	40	€2·51	28	€0·00	1,217	€2·73	
Pitavastatin 2-4mg	0·1%	3	€10·28	28	€0·12	91	€11·16	
Low intensity								
Pravastatin 10-20mg	0·1%	15	€3·92	28	€0·01	456	€4·25	
Lovastatin 20mg	0·1%	20	€1·60	28	€0·00	608	€1·74	
Fluvastatin 20-40mg	0·2%	30	€4·75	28	€0·01	913	€5·16	
No statin	3·3%	0					€ 0·00	
**Weighted average**							€14·22	

Table S12: ACE Inhibitor treatment unit cost and usage

	Usage	Mg per day	Unit Cost (per pack)	Pack size	Cost per mg	Mg per cycle	Total cycle cost	Source
Ramipril 2·5mg	43·7%	2·5	€3·10	56	€0·022	76	€1·68	SECURE.[1] BotPlus,[7] accessed 24th May 2023.
Ramipril 5mg	26·9%	5·0	€3·10	8	€0·022	152	€3·37	
Ramipril 10mg	7·5%	10·0	€6·20	28	€0·022	304	€6·74	
Enalapril 2·5mg	3·1%	2·5	€2·69	500	€0·002	76	€0·16	
Enalapril 5mg	4·0%	5·0	€5·37	500	€0·002	152	€0·33	
Enalapril 10mg	2·4%	10·0	€1·60	60	€0·003	304	€0·81	
Enalapril 20mg	2·1%	20·0	€1·03	28	€0·002	608	€1·12	
Perindopril 4mg	2·2%	4·0	€3·46	30	€0·029	122	€3·51	
Perindopril 8mg	5·4%	8·0	€6·91	30	€0·029	243	€7·01	
Lisinopril 5mg	1·6%	5·0	€2·18	60	€0·007	152	€1·11	
Lisinopril 20mg	1·0%	20·0	€4·07	28	€0·007	608	€4·42	
**Weighted average**							€2·73	

Table S13: ASA treatment unit cost and usage

	Mg (per day)	Unit cost	Pack size	Cost per mg	Mg per cycle	Total cycle cost	Source
Aspirin 100mg	100	€2·33	100·00	€0·0002	3,042	€0·71	SECURE.[1] BotPlus,[7] accessed 24th May 2023.

Table S14: Health state resource use (all values per patient)

GP monthly visits		Source
'No further event'	0·17	8
'Post-reinfarction'	0·17	
'Post-stroke'	0·57	9
**Cardiologist number of monthly visits**		
'No further event'	0·17	8
'Post-reinfarction'	0·17	
'Post-stroke'	0·47	9

Table S15: **Societal Perspective- Caregiver Productivity loss**

Cardiovascular Event	Number of hours of care per week†	Time	Monthly caregiver productivity loss per patient
Severe MI	24·5	First year post-reinfarction	€540·19
Severe MI	0	Post first-year	€0·00
Mild MI	0	First year post-reinfarction	€0·00
Mild MI	0	Post first-year	€0·00
Severe stroke	40·0	First year post-stroke	€577·09
Severe stroke	40·0	Post first year	€577·09
Mild stroke	0	First year post-stroke	0
Mild stroke	0	Post first year	0

†It was assumed that 50% of these hours would replace work time; MI, myocardial infarction

Scenario Analyses

A series of deterministic scenario analyses were performed to assess the robustness of the results when different inputs or assumptions were varied.

Scenario 1: Parametric Survival Curves: Second Best-Fitting Distributions

The best-fitting distributions for each of the parametric survival curves in the base case were chosen based on a combination of the AIC/BIC and clinical plausibility. As a scenario analysis, each of the distributions were changed to the second-best fitting distribution according to the AIC/BIC values, to determine the impact on the results. The log-logistic distribution was selected for all distributions (Figure S5 to S8).

Figure S5: Time to reinfarction modelled via Log-logistic functions

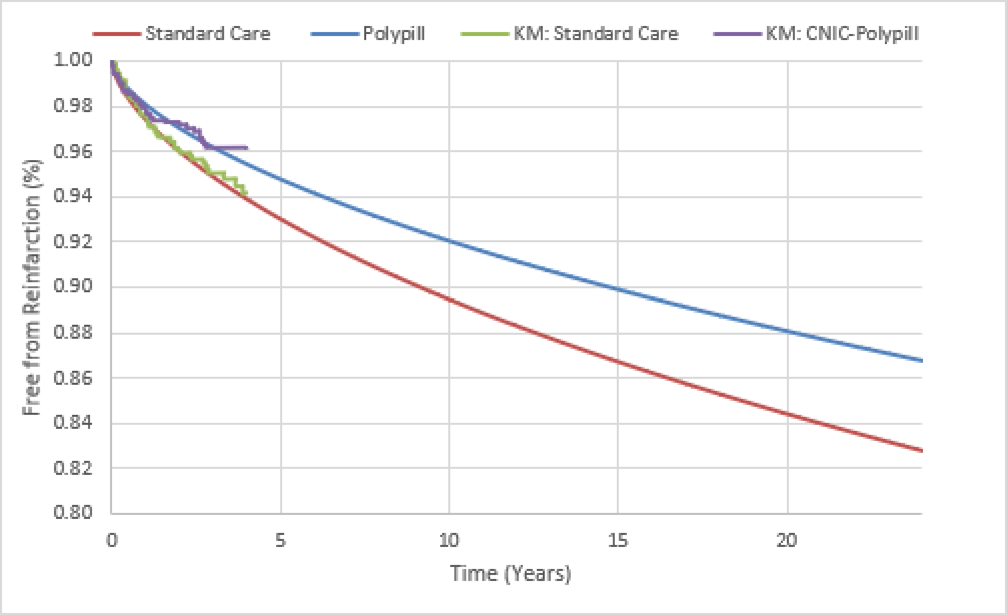

Figure S6: Time to stroke modelled using Log-logistic functions

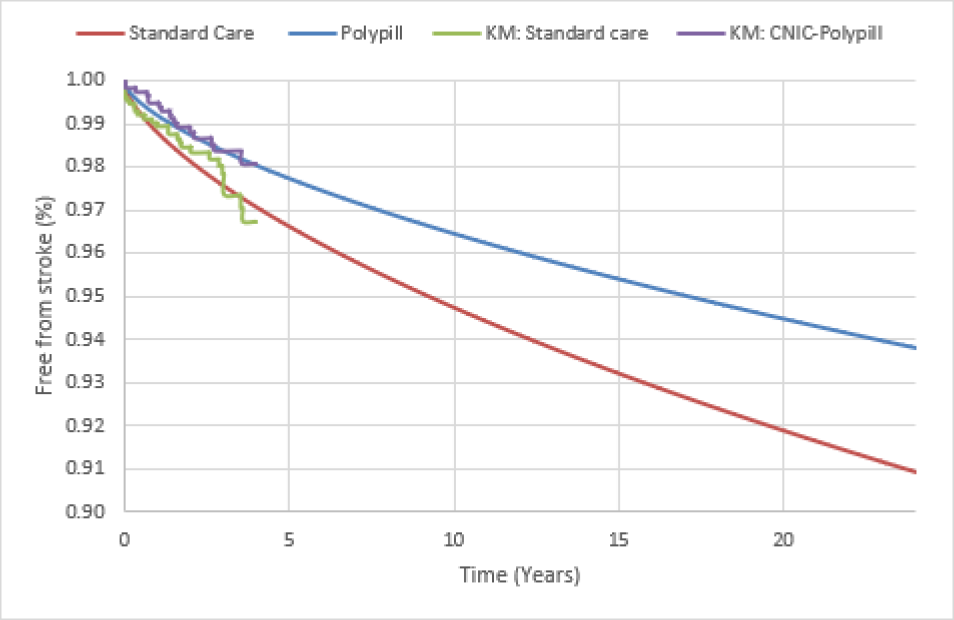

Figure S7: **Time to CVD death modelled using Log-logistic functions**

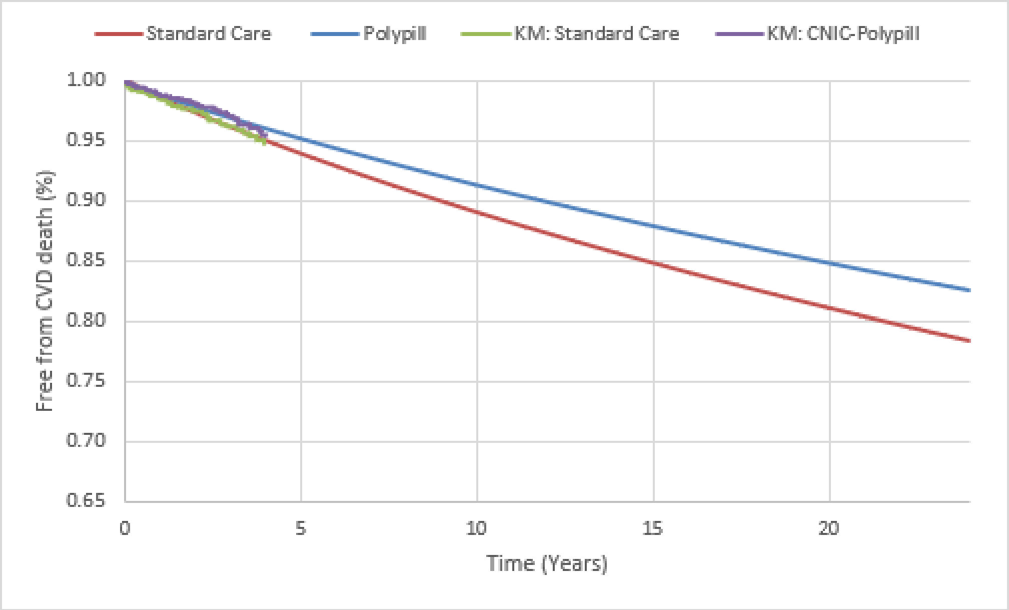

Figure S8: **Time to non-CVD death modelled using Log-logistic functions**

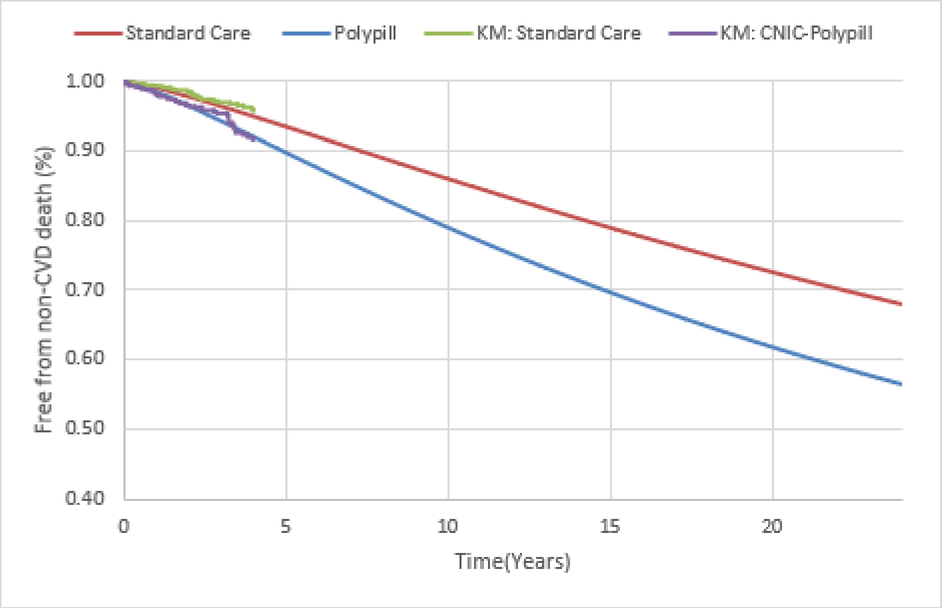

Scenario 2: Treatment Waning Effect

The base case analysis assumed that there would be no treatment waning effect associated with the CV-Polypill. Two scenarios were run to test this assumption. In the first scenario, an instant treatment waning effect was applied from year four (i.e. at the completion of the SECURE trial). In this scenario, the probability of reinfarction, stroke and death were assumed to be equivalent to standard of care from year four onwards. In the second scenario, a linear gradual treatment waning effect was applied from year four to year ten. The waning effect in both scenarios was applied to the four parametric survival models used to determine the transition of patients from the 'no further event' health state. No treatment waning effect was applied once patients had left this health state.

Scenario 3: Percentage of patients who remain on treatment following a cardiovascular event

It is currently uncertain whether patients would remain on treatment with the CV-Polypill or standard care following reinfarction or stroke. In the base case analysis, it was assumed that treatment would remain unchanged after reinfarction or stroke in both arms of the model. This scenario assumed that 50% of patients in both arms would discontinue active treatment following reinfarction or stroke. This only impacted costs and had no impact on efficacy or health-related quality of life (HRQoL) which is assumed equal regardless of treatment arm.

Scenario 4: Societal Perspective

The base case analysis took the perspective of the Spanish National Health System. However, a scenario incorporating a broader societal perspective was also run to incorporate caregiver productivity based on the requirement for caregivers to reduce work hours when caring for a patient who has experienced a stroke or reinfarction. It was assumed that one caregiver would be required to substitute 12·25 hours of work a week in the first year following reinfarction and 20 hours post-stroke until the patient (with the remainder of caregiver time taken from 'personal hours' such as evenings and weekends). Further detail on these calculations is provided in Table S15.

Productivity costs were not applied directly to patients because the mean age in the model (76 years) was above retirement age and, therefore, it was assumed that the population within the simulated cohort were not actively working.

Scenario 5: Time Horizon Set to Match Follow-Up of SECURE Trial

The model base case was completed over a lifetime time horizon to capture the long-term costs and benefits associated with the CV-Polypill, in accordance with international health technology assessment agency guidelines. [10] A scenario was run whereby this was changed to a four-year time horizon (i.e. the follow-up length of the SECURE trial).

Scenario 6: Utilities Provided by the SECURE Trial Used

Utility values obtained from the SECURE trial were not used in the base case as they were above the general population norms for Spain and, therefore, literature values were deemed more appropriate. A scenario analysis was run using values obtained from the SECURE trial. A general linear beta regression model, with a logit link function, was used to estimate the average HRQoL at baseline, while adjusting the dependent variable for baseline characteristics. This utility value was used for the 'no further event' health state. This meant the adjustment factor was 1·0013 (0·858/0·857) and, therefore, individuals were assigned general population norms in the 'no further event' health state to ensure the utility of patients in the model could not be greater than the general population norms. It was not possible to use HRQoL values obtained from the SECURE trial in other health states as the utility data was only collected at baseline and two years.

Scenario 7: No Treatment Effect

This scenario assumed that the treatment effect coefficients within all of the parametric survival analyses were set to zero (i.e. there was no treatment effect) - this was tested because the treatment-specific coefficients were not significant.

Scenario 8: Standard Care Dosage Set to Match the CV-Polypill Arm

This scenario assumed the treatment dosages within the standard care arm matched the CV-Polypill of the SECURE trial exactly. Therefore, patients could only receive treatment with aspirin, ramipril and atorvastatin. Please note that this scenario impacted costs only.

Additional base case analysis results

Table S16: Discounted cost breakdown – per person

	Polypill	Standard Care	Difference
Pharmaceutical costs	€1,706·86	€1,949·09	-€242·23
Healthcare contact costs pre- secondary event	€3,331·36	€3,243·75	€87·62
'Post-reinfarction' costs: immediately after event	€423·90	€517·42	-€93·53
'Post-reinfarction' ongoing costs	€104·13	€128·75	-€24·62
'Post-stroke' costs: immediately after event	€162·38	€239·11	-€76·73
'Post-stroke' ongoing costs	€167·35	€247·42	-€80·07
Urgent revascularisation costs	€443·34	€438·73	€4·61
Cardiovascular death	€1,095·19	€1,374·67	-€279·48
Non-cardiovascular death	€3,510·93	€3,398·02	€112·90
Total	€10,945·45	€11,536·97	-€591·53

Figure S9: Accrued health state occupancy

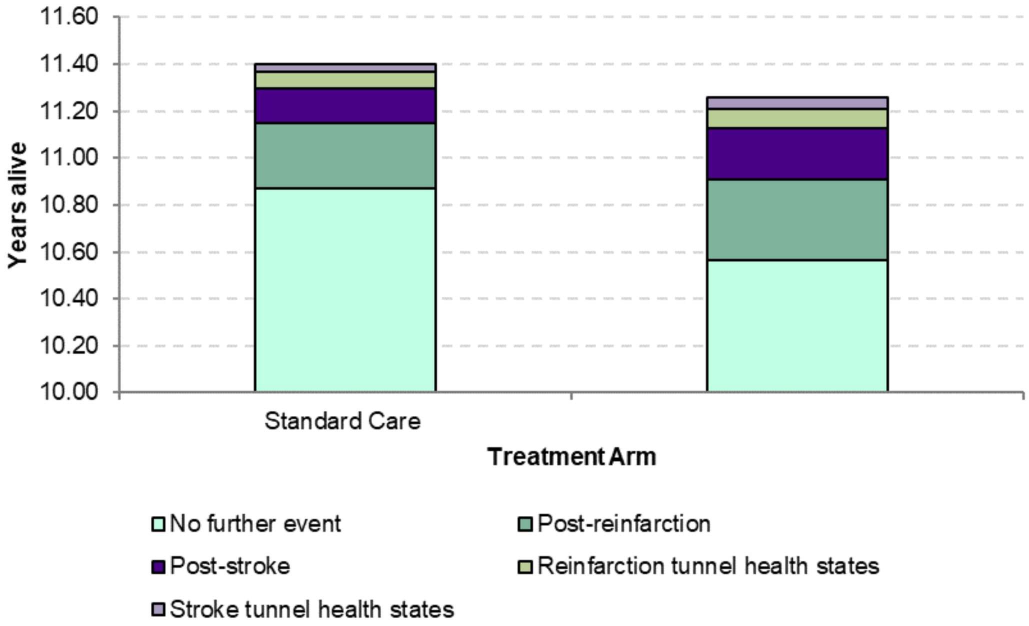

References

1. Castellano JM, Pocock SJ, Bhatt DL, et al. Polypill Strategy in Secondary Cardiovascular Prevention. *New England Journal of Medicine*. 2022;387(11):967-977. doi:10.1056/NEJMoa2208275
2. National Institute for Health and Care Excellence. Ticagrelor for the treatment of acute coronary syndromes https://www.nice.org.uk/guidance/ta236/documents/acute-coronary-syndromes-ticagrelor-astrazeneca4
3. Rabier H, Serrier H, Schott A-M, et al. Economic valuation of informal care provided to people after a myocardial infarction in France. *BMC health services research*. 2019;19:1-8.
4. Lee JH, Huber JC, Jr. Evaluation of Multiple Imputation with Large Proportions of Missing Data: How Much Is Too Much? *Iran J Public Health*. Jul 2021;50(7):1372-1380. doi:10.18502/ijph.v50i7.6626
5. StataCorp. *Stata Statistical Software: Release 17. College Station, TX.* 2021.
6. School of Health and Related Research UoS, UK NICE DSU Technical Support Document 14: Survival Analysis for Economic Evaluations Alongside Clinical Trials - Extrapolation with Patient-Level Data. https://www.ncbi.nlm.nih.gov/books/NBK395885/pdf/Bookshelf_NBK395885.pdf
7. BOTPLUS. https://botplusweb.farmaceuticos.com/
8. Escobar-Cervantes C, Villa G, Campos-Tapias I, et al. Achieving Lower LDL-C Levels After a Recent Myocardial Infarction Might Be Associated with Lower Healthcare Resource Use and Costs in Spain. *Advances in Therapy*. 2022;39(8):3578-3588.
9. van Mastrigt G, van Heugten C, Visser-Meily A, Bremmers L, Evers S. Estimating the burden of stroke: two-year societal costs and generic health-related quality of life of the Restore4Stroke Cohort. *International Journal of Environmental Research and Public Health*. 2022;19(17):11110.
10. National Institute for Health and Care Excellence. NICE health technology evaluations: the manual https://www.nice.org.uk/process/pmg36/resources/nice-health-technology-evaluations-the-manual-pdf-72286779244741
